# Automated Detection of Hypertension Using Physiological Signals: A Review

**DOI:** 10.3390/ijerph18115838

**Published:** 2021-05-29

**Authors:** Manish Sharma, Jaypal Singh Rajput, Ru San Tan, U. Rajendra Acharya

**Affiliations:** 1 Department of Electrical and Computer Science Engineering, Institute of Infrastructure Technology Research and Management, Ahmedabad 380026, India; jaypal.rajput.18pe@iitram.ac.in; 2 National Heart Centre, Singapore 639798, Singapore; tanrsnhc@gmail.com; 3 Department of Electronics and Computer Engineering, Ngee Ann Polytechnic, Singapore 639798, Singapore; aru@np.edu.sg; 4 Department of Bioinformatics and Medical Engineering, Asia University, Taichung 41354, Taiwan; 5 Department of Biomedical Engineering, School of Science and Technology, SUSS, Singapore 599494, Singapore

**Keywords:** hypertension, ECG signal, HRV signal, BCG signal, PPG signal, deep learning, CNN, ANN, RNN, supervised machine learning, HT ECG signal classification

## Abstract

Arterial hypertension (HT) is a chronic condition of elevated blood pressure (BP), which may cause increased incidence of cardiovascular disease, stroke, kidney failure and mortality. If the HT is diagnosed early, effective treatment can control the BP and avert adverse outcomes. Physiological signals like electrocardiography (ECG), photoplethysmography (PPG), heart rate variability (HRV), and ballistocardiography (BCG) can be used to monitor health status but are not directly correlated with BP measurements. The manual detection of HT using these physiological signals is time consuming and prone to human errors. Hence, many computer-aided diagnosis systems have been developed. This paper is a systematic review of studies conducted on the automated detection of HT using ECG, HRV, PPG and BCG signals. In this review, we have identified 23 studies out of 250 screened papers, which fulfilled our eligibility criteria. Details of the study methods, physiological signal studied, database used, various nonlinear techniques employed, feature extraction, and diagnostic performance parameters are discussed. The machine learning and deep learning based methods based on ECG and HRV signals have yielded the best performance and can be used for the development of computer-aided diagnosis of HT. This work provides insights that may be useful for the development of wearable for continuous cuffless remote monitoring of BP based on ECG and HRV signals.

## 1. Introduction

In adults, hypertension (HT) is diagnosed when repeated office measurement of systolic blood pressure (SBP) is ≥140 mmHg, or diastolic blood pressure (DBP) is ≥90 mmHg [1]. HT can be classified into different categories based on the office measurement (Table 1) [1]. HT increase the force exerted by by the blood against the inner walls of the arteries, which transport oxygen-rich blood pumped out of the heart to the rest of the body [2]. As such, chronic HT can inflict damage to various vital organs of the body, such as lung, brain, heart, and kidneys [2]. The World Health Organization estimates that nearly 1.3 billion people suffered from HT in 2015 globally, and less than 20% received management [2]. HT is largely asymptomatic, but symptoms can sometimes occur, including headaches, panic attacks and dizziness.

### 1.1. ECG Signals and Blood Pressure (BP) Measurements

**Figure 1:**
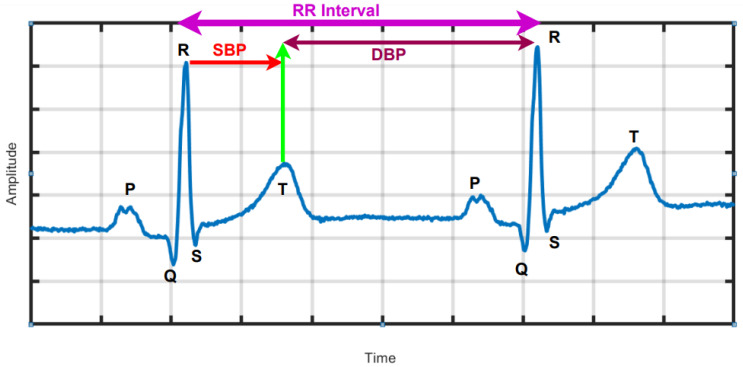
ECG waveform with standard intervals. Correlations have been found between systolic (SBP) and diastolic blood pressure (DBP) measurements and morphological data in the corresponding indicated epochs. The Figure is generated from PTB database (subject no. 14).

The electrocardiogram (ECG) records the electrical potentials on the body surface that originate from heart, and the signals can provide information on the rhythm as well as structure and function of the heart [3,4,5,6]. Using advanced analysis, the ECG signals in HT subjects can be correlated to BP measurements and even discriminate for higher clinical risk [7,8,9,10]. In HT, the heart observes more force and over time becomes hypertrophied, which induces the ECG. Figure 1 is a graphical depiction of a typical normal ECG waveform, which comprise the P wave, QRS complex and T wave representing atrial depolarization, ventricular depolarization and ventricular re-polarization, as well as standard ECG intervals, including RR interval, PR interval, QT interval and lengths of the PR and ST segments. In [7], associations were found between SBP and DBP and changes in the ECG at two intervals delineated by the peak of the R wave to the middle of the T wave and the mid of the T-wave to the peak of the R wave as indicated in Figure 1, respectively, using machine learning (ML) [11,12].

### 1.2. HRV Signal

The temporal variation of sequential heartbeats (RR intervals) is termed HRV [13]. From the ECG signal, R-peaks are first extracted and then HRV is deduced using computer programming based on the difference in RR intervals (Figure 1). HRV reflects the activity of automatic nervous system and provides a window into the cardiac sympathetic and parasympathetic activities, which have significant physiological impact on heart rate rhythm and contractile function [13]. HRV measurement is non-intrusive easy to perform and results are reproducible. Importantly, it confers both diagnostic and prognostic implications for wellness and cardiac disease [14,15]. High HRV is associated with normal subjects and reduced HRV may be pathological. HRV can be analyzed over either long or short durations [14]. Long duration analysis encompasses activity throughout the day and night (24 h analysis), where as short-duration analysis uses only five-minutes of HRV data. In HT patients, HRV is affected by the presence of cardiovascular risk factors. In depressed patients with HT, HRV is associated with vascular cardiac and renal target organ damage [13,16]. The long-term (24-h) HRV is useful in the diagnosis of severe HT conditions. An increased sympathetic activity saturate the ability to modulate heart rate, hence HRV is depressed. To identify severe HRV or high-risk HRV, standard deviation of NN intervals should be less than 50 to 70 msec and HRV triangular index is less than 20 units. Similarly, the NN interval duration is 7.8 msec [17]. In summary, HRV is a simple non-invasive method which can be used to assess the cardiovascular system [18].

### 1.3. Photoplethysmography (PPG Signal)

PPG uses low-intensity infrared (IR) light sensor to detect the amount of light absorbed by or reflected from tissues supplied by the blood vessels. It produces photo electric signal either transmissive or reflective, which reflect the pulsatile blood volume in the area covered by sensors [19,20]. The PPG signal contains information about the arterial and venous circulatory system [19,20]. The PPG is correlated with and has been applied to the measurement of heart rate, BP, and blood oxygenation there by providing clinically useful information for physiological monitoring.

### 1.4. Ballistocardiogram (BCG) Signal

BCG measures the whole body motion in terms of displacement, velocity, and acceleration in response to the cyclical ejection of blood from the heart [21]. It reflects the sum of factors linked to heart and blood vessel function, and used to diagnose various cardio vascular diseases [21].

In this paper, we reviewed PPG, BCG, ECG and HRV signals related computer-aided diagnosis systems developed for the arterial HT. To the best of our knowledge this is the first review to provide unique ranges for nonlinear features for healthy control (HC), low-risk hypertension (LRHT), and high-risk hypertension (HRHT) ECG classes.

## 2. Methods and Material Used in Article Searching

This review was carried out based on the PRISMA model for the period between 09 October 2008 and 31 March 2021 [22]. Science Direct, Web of Science, Google Scholar, PubMed, IEEE Explorer, and ResearchGate databases were systematically searched using the following keywords: (“hypertension”, “ECG and hypertension”, “HRV or hypertension”), “photoplethysmography (PPG) and hypertension”, “photoplethysmography (PPG) and ballistocardiogram (BCG)”, “unsupervised machine learning and hypertension”, “supervised machine learning and hypertension”, “detection of hypertension”, “convolution neural network (CNN)”, “Hypertension and Convolution neural network”, “machine learning and hypertension”, “hypertension and deep learning”, “recurrent neural network (RNN)”, “support vector machine (SVM)”, “automated detection of hypertension”, “detection of hypertension using ECG signal”, “hypertension or HRV signal”, “systolic blood pressure (SBP)”, “diastolic blood pressure (DBP)”, “electrocardiogram (ECG)”, “systolic blood pressure and hypertension”, “diastolic blood pressure and hypertension”, and “physiological signal and hypertension”. We identified 250 research articles containing these keywords on initial screening. Science Direct = 50, Web of Science = 40, Google Scholar = 40, PubMed = 50, IEEE Explorer = 50, and ResearchGate = 20. Among these, 103 duplicate articles were omitted.

We further excluded non-English articles and works that were not explicitly designed for diagnosis of HT.

**Figure 2:**
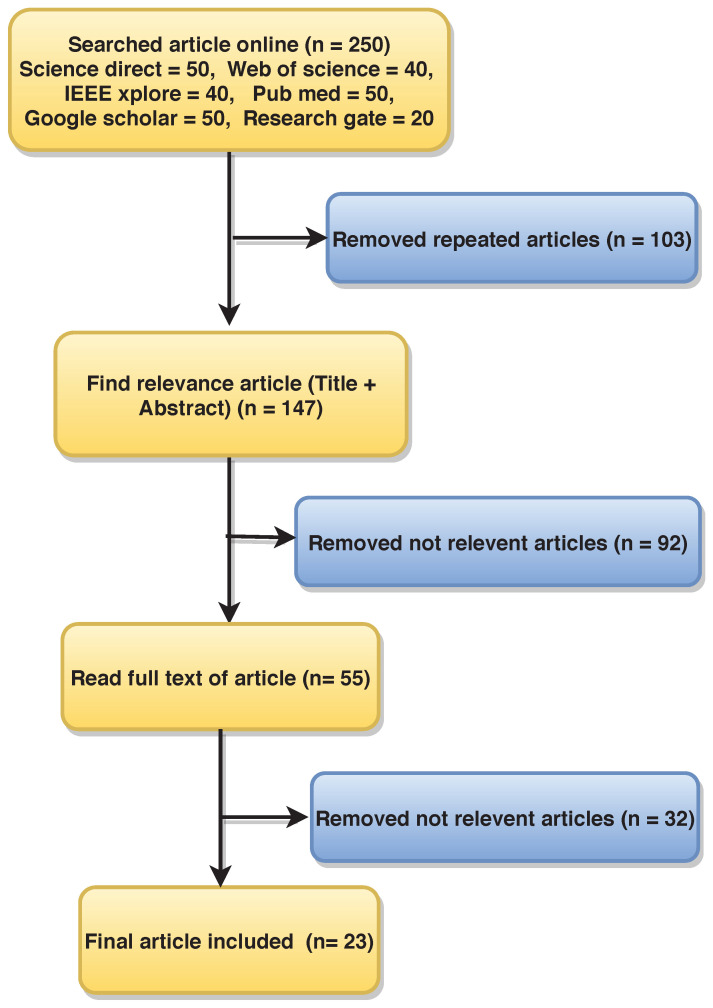
Flow diagram of the article selection process using PRISMA methodology.

Finally, 23 articles were selected for this review. Figure 2 shows the flow diagram of article selection, where n is the number of articles.

## 3. Databases

The ECG, HRV, BCG, and PPG databases are used to develop an automated HT systems and are summarized in Table 2, Table 3 and Table 4. The relative percentages of the different signals used are: ECG = 30.43%, PPG = 17.39%, BCG = 8.69%, and HRV = 43.47%. The most common databases are based on HRV and ECG signals.

### 3.1. ECG Signal Database

The open-source “Smart Health of Accessing the Risk of Events via ECG Signal” database (SHAREE) used by [2,8,23,24,25] comprised of 24-hour ECG recorded using three ECG leads (II,III, and V5) at 128 Hz sampling frequency.

### 3.2. ECG Derived HRV Signal Databases

HRV signals extracted from SHAREE were used in [13,16,26]. In [27], HRV signal derived from 10-min ECG recordings from 568 subjects were studied.

One group studied 113 HRV signals [28], and later an expanded 185-sample HRV dataset [29], derived from 7-min Lead II ECG recordings sampled at 500 Hz collected at the same center.

HRV signals were obtained from 7- to 9-hour ECG records (sampling frequency 200 Hz) from 24 subjects in [30]. In [18], a Kubios HRV analyzer was used to derive HRV from 5-min Lead II ECG recordings.

Seventy-one HRV signals derived from 300-s ECG recordings were studied in [31].

Ten minute 12-lead ECG signals sampled at 200-Hz in 97 subjects were used to derive the HRV dataset in [32].

### 3.3. BCG-Derived HRV Signals Database

BCG recordings sampled at 100 Hz were used to derive HRV signals for 18 subjects in [33]. In [21], HRV signals were extracted from 67 normal and 61 HT BCG signals sampled at 100 Hz with 16-bit resolution.

### 3.4. PPG Signal Database

In two different studies by the same group, 120-second PPG recordings (sampling frequency 125 Hz) from Multiparameter Intelligent Monitoring in Intensive Care Database (MIMIC) were used [34,35]. In [20], the same authors studied 124 180-second PPG signal recordings ( sampling frequency 1kHz) collected at the same hospital. In [15], HRV signals were extracted from 43 PPG recordings sampled at 64 Hz with 8-bit resolution. Twenty PPG signals encompassing 1536 hours of data in normal and HT subjects were used to derive HRV in [36].

## 4. Pre-Processing of ECG Signals

### 4.1. Normalization

Rajput et al. [8,23] used Z-score normalization method for amplitude scaling of ECG signal. The Z-score is the difference between the mean and actual ECG signal divided by the standard deviation of the ECG signal. Similarly, Liang et al. [20,34,35] and Liu et al. [21] used Z-score score normalization to normalize the amplitudes of PPG and BCG signals, respectively.

### 4.2. Segmentation

Segmentation is used to convert long-duration (e.g., 24-h) signals into short-duration ones requiring shorter computation time for downstream analysis. Soh et al. [2] also segmented the ECG signals of 139 HT subjects into 69,500 segments with, each sample size of 2000 samples.

In [8,23,26] 24-hour ECG signals from (SHAREE) were segmented into 5-and 2-min segments, respectively, for analysis.

In [13], 8-hour ECG signals from SHAREE were segmented into 4, 2, and 1-h as well as 30, 20, 10, and 5-minutes segments for analysis. The performance of 20 and 5-min ECG signals performed better than long-duration ECG signals. Melillo et al. [16] segmented 10-h ECG signals into 5min segments.

### 4.3. Signal Filtering

Low and high-frequency noise signals, generated during the recording of ECG signal, may affect the interpretation [39]. ECG signal noise can be induced by electrode contact noise, electromyogram, channel noise, baseline wander, and power line interference [39]. It is important to remove noise from the ECG signal to obtain higher classification performance for which various methods are available. Ni et al. [30] used Savitzky-Golay filtering to remove noise from the digitized ECG signal, while Soh et al. [2] used discrete wavelet transform (DWT). For the removal of noise from PPG signals, Liang et al. [20,34,35] applied Chebyshev II band-pass filter with a frequency range of 0.5–10 Hz.

### 4.4. Re-Sampling

Poddar et al. [28] employed BIOPAC 4.0 software to extract the RR tachographs from ECG signals. The tachographs contained samples that were unevenly placed due to beat-to-beat variation of RR intervals. Re-sampling at a frequency of 4 Hz was performed to preserve the uniformity across the entire length of tachograph data.

### 4.5. Discrete Wavelet Transform (DWT)

Liu et al. [21] applied DWT to decompose the BCG signal into multiple time-frequency resolutions, through which the details of the signal could be clearly described in time and frequency domains jointly [9,10,11,40,41,42]. DWT decomposes the BCG signal into detailed and approximate components, via iterative low and high-pass filtering [43,44,45,46,47].

### 4.6. Continuous Wavelet Transform (CWT) Used for PPG Signal Transformation

The PPG signals are converted into two dimensional images called scalograms and fed as input to the convolutional neural network (CNN) for automated detection of HT PPG signals [31,34].

## 5. Features Extracted in the Review Studies

### 5.1. HRV Features

#### 5.1.1. HRV Time-Domain Parameters

HRV parameters are extracted from RR intervals and can be categorized into, short-term variation (STV) and long-term variation (LTV) in the time domain [14]. LTV exhibits slower, and STV faster fluctuation. RR intervals have the following intrinsic features: intervals between normal heart beats of ECG signal (NN); standard error of NN intervals (SENN); standard deviation of differences between adjacent NN intervals (SDSD); root mean square of successive differences between NN intervals (RMSSD); the number of successive NN intervals that differs from each other by >50 ms of the whole recording (NN50); and the percentage of successive NN intervals that differs by >50 ms of the whole recording (pNN50%) [14].

#### 5.1.2. HRV Frequency-Domain Parameters

Fast Fourier transform (FFT) decomposes the RR intervals into their frequency constituents, which can be classified as very low frequency (VLF), low frequency (LF), and high frequency (HF). Total power (TP) is a short-term estimate of total power of power spectral density in the range of frequencies between 0 and 0.4 Hz. However, TP mainly reflects level of the autonomic nervous activities (both parasympathetic (PNS) and sympathetic (SNS)) and humoral (hormonal) effects and circadian rhythm as well as ANS’s activity. Generally decrease in TP is observed in individual under chronic stress or with disease [14,48]. HF (0.15 to 0.4 Hz) and LF (0.04 and 0.15 Hz) reflect the modulatory effects of parasympathetic and sympathetic activity, respectively, on the heart rate. Accordingly, the ratio of LF to HF represents the sympathovagal balance. VLF( 0 to 0.04 Hz ) reflects the vascular response associated with mechanisms caused by negative feelings [48,49].

### 5.2. Features of BCG Fluctuation

Cardiac mechanical operations modulate fluctuation pattern of the BCG signal, and cardiovascular disease such as HT can be identified by evaluating the pattern of fluctuation pattern [21]. As the BCG signal commonly includes noise from body motion and the signal acquisition system itself, it is important that BCG fluctuation features acquired be noise-sensitive. Four noise-insensitive features are used: zero-crossing rate (ZCR); average cumulative amplitude change (ACAC); average number of extreme points (ANEP); and average signal turns count(ASTC) [21].

### 5.3. Non-Linear Features Extracted from ECG and HRV Signal

The non-linear features that can be computed for ECG and HRV signals include: sample entropy (SeEn) [2,23], approximate entropy (ApEn) [2,27,28,30], renyi entropy (ReEn) [2,13], wavelet entropy (WlEn)[2,23], detrended fluctuation analysis (DFA) [13,16,21,33], correlation dimension (CD) [16,38], Lempel-Ziv complexity (LZ) [27], recurrence plot (RC) [16], Poincare plot (PP) [16,28], empirical mode decomposition (EMD) [24], signal fractal dimension (SLFD), Log energy (LOGE) [8], higher-order spectra (HOS), HOS cumulants, fuzzy entropy, Kolmogorov-Sinai entropy, modified multiscale entropy, Shannon entropy, permutation entropy, and Tsallis entropy [2].

### 5.4. Feature Selection, Reduction, and Ranking

Feature selection helps to select most relevant features which can be used to distinguish between normal versus HT classes. Various feature reduction techniques employed include principal component analysis (PCA) [13,31], marginal Fisher analysis (MFA), linear discriminant analysis (LDA), temporal pyramid pooling method (TPPM) [13], and independent component analysis.

The features are organized and presented to the given classifier based on their ranking one-by-one until the highest performance is obtained.

Student’s *t*-test [2,8,13,16,21], Bhattacharya, Wilcoxon, and receiver operating characteristics (ROC) are the mostly used feature-ranking techniques. Multi-factor analysis of variance (MANOVA) and the chi-square test [16] are utilized as the feature selection methods for choosing the highly discriminant features to the classifier.

## 6. Computer-Aided Diagnosis Methods

**Figure 3:**
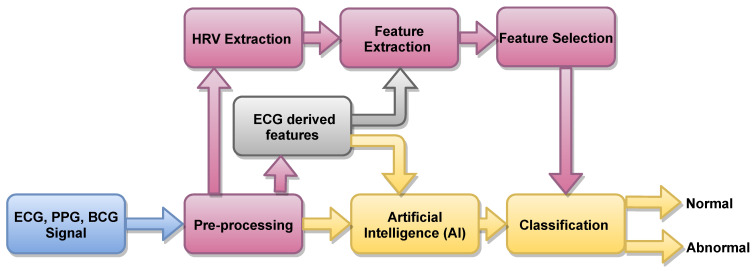
Proposed automated system to detect HT ECG signals.

In Figure 3, an outline of methods based on artificial intelligence (AI) is presented. About 82.6%, 13.04% and 4.34% of authors used ML the statistical software SPSS, and other traditional methods, respectively to detect HT ECG signals automatically. ML-based techniques are robust and accurate.

### Hypertension Diagnosis Index (HDI) [8]

Rajput et al. [8] developed an HDI using selected features to accurately discriminate low-risk HT (LRHT) and high-risk HT (HRHT) with a single numeric value.

The orthogonal wavelet filter bank is used for five-level wavelet decomposition. Signal fractal dimension (SLFD) and log energy (LOGE) features were then computed from the decomposed coefficients. All 12 sub-bands of ECG signal were ranked by the Student’s t-test ranking method. High-ranking feature sub-bands (SUB) were used to develop HDI (Equation (1)). In Equation (1), various composite features were experimentally merged to achieve the optimal difference between the two groups.

(1) $$\begin{array}{c} HDI=6-(3\times LOGE_{SUB2}+4\times LOGE_{SUB3}+SLFD_{SUB6})\\ -15\times (SLFD_{SUB2}+SLFD_{SUB3}+SLFD_{SUB4}), \end{array}$$

where $LOGE_{SUB2},LOGE_{SUB3}$ denoting sub-bands (second and third) of log energy, while $SLFD_{SUB2},SLFD_{SUB3},SLFD_{SUB4},SLFD_{SUB6}$ are sub-bands (second, third, fourth, and sixth) of signal fractal dimension feature.

## 7. Proposed Work after Understanding Review Studies

**Figure 4:**
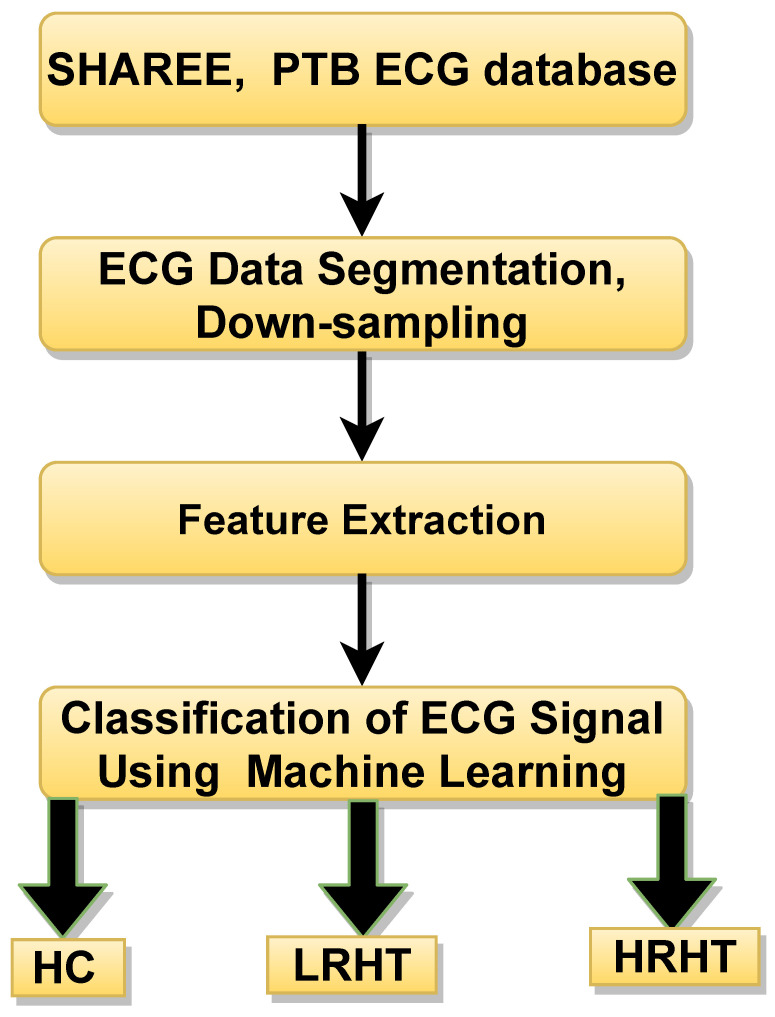
Workflow of proposed methods for HT diagnosis using ECG signals. HC represents healthy control, LRHT, low-risk hypertension, and HRHT is high-risk hypertension.

An outline of the proposed work is shown in Figure 4. In this work, we used the public database used by authors in [23,24]. A total of 3694 ECG segments were obtained from (SHAREE, and PTB data base) [23,24]. The LRHT have 3172 (SHAREE database), HRHT (SHAREE database) have 442, and HC class have 80 (PTB database) ECG signals segmented in to 2 minute duration. A single ECG-lead V5 have been chosen from both databases. To match the sampling frequency of SHAREE and PTB database, we have down-sampled the data obtained from PTB database. The details of the extracted features are discussed below:

### 7.1. Features Extracted to the Proposed Work

#### 7.1.1. Sample Entropy (SeEn)

It is a measure of uncertainty in the non-linear signal [2,23]. SeEn can be computed as;

$$SeEn=-\log(\frac{a}{b}),$$

where a is the l+1 length of vector, and b is the length *l* of vector with l=2 [23].

#### 7.1.2. Approximate Entropy (ApEn)

It is an approximation technique which is useful to measure homogeneity and complexities in time-series data containing noise because of its stability to distinguish closely linked stochastic processes. It is effective in short data intervals to differentiate between chaotic and noisy time-series data [2,27,28,30]. For finite length N, ApEn is computed as:

$$ApEn\left(m,r\right)=\Phi {\left(r\right)}^{m}-\Phi {\left(r\right)}^{m+1},$$

Φ(r) is a vector, while *m* = 2. The tolerance value *r* = 0.20 lies between 0.15 to 0.25 [28].

#### 7.1.3. Renyi Entropy (ReEn)

It is used to measure the spectral intricacy of time-series signals and generalized as the Shannon entropy [2,13]. ReEn is computed as :

$$ReEn\left(K\right)=-\frac{\alpha }{1-\alpha }\sum \left(\log p_{j}^{\alpha }\right),$$

where *K* = discrete random variable, α is order (α≥2) of ReEn, $p_{j}$ = denoted the total spectral-power [24,50].

#### 7.1.4. Wavelet Entropy (WlEn)

It is used to calculate the degree of disorder in an ECG signals [2,23,40,51].

$$WlEn=-\sum_{i<0}\left(P_{i}\times \ln\left(P_{i}\right)\right),$$

where *i* is a resolution level and $P_{i}$ is probabilities with respect to *i* [52].

#### 7.1.5. Log Energy (LOGE)

It is the logarithm of an energy of the ECG signal. The mathematical expression of LOGE is given below [53].

$$LOGE_{r}=log\left(\sum_{n}{m_{r}\left(n\right)}^{2}\right),$$

where $LOGE_{r}$ is define the log energy of *r*th time-series, and the amplitude of *n*th sample of *r*th time-series is $m_{r}\left(n\right)$.

#### 7.1.6. Signal Fractal Dimension (SLFD)

It is used to measure similarity and complexity in physiological signals. It is the ratio of fractal pattern with respect to which it is measured [53]. It is given by:

(2) $$SLFD=\frac{log(A_{l})}{log(\frac{1}{l})}.$$

#### 7.1.7. Hurst Exponent (HE)

It is a measure of repeat-ability [54]. The generalize equation of HE is as follows:

$$HE=\frac{Log(\frac{A}{B})}{Log(Y)}.$$

Here, Y represents the length of time-series data, while $\frac{A}{B}$ denoted the re-scaled range value. The difference of maximum and minimum value of mean is considered as A. However, B represents the standard deviation.

#### 7.1.8. Largest Lyapunov Exponent (LLE)

LLE is used to identity chaos in the physiological signal [55].

#### 7.1.9. HOS Bispectrum (HOSB)

A third-order statistics computation is know as an HOS bispectrum [56]. The Fourier transform of third order cumulant is the bispectrum $S_{3}\left(\omega_{1},\omega_{2}\right)$ of a signal [57]. In a two-dimensional frequency plot, the bispectrum shows the cross-correlation between frequency components. Hence, the HOSB is define as:

(3) $$S_{3}\left(\omega_{1},\omega_{2}\right)=X\left(\omega_{1}\right)X\left(\omega_{2}\right)X^{*}\left(\omega_{1}+\omega_{2}\right).$$

However, HOSB represents the triple product of its two frequencies. To define principle region or non-redundant region sufficient condition $\omega_{2}\geq$ 0, $\omega_{2}\geq \omega_{1}$, $\omega_{1}+\omega_{2}$≤π must satisfied [56]. To analyze the bispectrum plots, different parameters namely moments, centroid, and entropies of the distribution may be extracted [56]. These features and parameters are as follows:

(a) Normalized bispectral entropy (NBE) [56,58]:
  (4) $$NBE1=-\sum_{n}q_{n}\log q_{n},$$
  where $q_{n}=\frac{\left|B(f_{1},f_{2})\right|}{\sum_{\Omega }\left|B\left(f_{1},f_{2}\right)\right|}$ and Ω represents the principle region.
(b) Normalized bispectral square entropy (NBSE) [58]:
  (5) $$NBSE=-\sum_{n}q_{n}\log q_{n},$$
  where $q_{n}=\frac{|B\left(f_{1},f_{2}\right){|}^{2}}{\sum_{\Omega }{\left|B\left(f_{1},f_{2}\right)\right|}^{2}}$. Both entropies are computed for parameters which have values between 0 and 1.
(c) A weighted center feature of bispectrum (WCOB) is described as [58]:
  (6) $$WCOB_{1m}=\frac{\sum_{\Omega (kB(k,l))}}{\sum_{\Omega B(k,l)}}$$
  (7) $$WCOB_{2m}=\frac{\sum_{\Omega (lB(k,l))}}{\sum_{\Omega B(k,l)}}.$$
  Here, *k* and *l* represents the frequency bin index in the principle region of bispectrum plot [58]. Similarly, some moments related features are given below:
(d) Bispectrum logarithmic amplitude feature [58]:
  (8) $$M_{1}=\sum_{\Omega }\log(|B\left(f_{1},f_{2}\right)\left|\right).$$
(e) Bispectrum sum of logarithmic amplitude of diagonal elements feature [58]:
  (9) $$M_{2}=\sum_{\Omega }\log(|B\left(f_{q},f_{q}\right)\left|\right).$$
(f) Bispectrum first-order spectral moments of amplitude of diagonal elements feature [58]:
  (10) $$M_{3}=\sum_{q=1}^{N}k\log(|B\left(f_{q},f_{q}\right)\left|\right).$$
(g) Bispectrum mean magnitude feature [58]:
  (11) $$mAmp=\frac{1}{L}\sum_{\Omega }\left|b\left(f_{1},f_{2}\right)\right|.$$
(h) Bispectrum phase entropy feature [58] :
  (12) $$Ph_{e}=\sum_{n}p\left(\Phi_{n}\right)\log p\left(\Phi_{n}\right).$$

Here, the number of points is represented by L of the principle region, Φ is the phase angle, and Ω refer the space of the region [58].

#### 7.1.10. Higher Order Spectral Cumulant (HOSC)

Obtaining the nonlinear dynamical characteristics of ECG signal using lower-order (first) of statistics is complex [57]. Therefore, higher order statistics, such as second, third and fourth, are widely used to analyze the ECG signal. Hence, HOS cumulant higher order statistics features are used in analysis of non-stationary ECG signals in various applications [57].

Let $\left\{r_{1},r_{2},r_{3}....,r_{q}\right\}$ is representing a zero mean random process for *q* dimensional multivariate. While $m_{1}^{r}$, $m_{2}^{r}$, $m_{3}^{r}$, and $m_{4}^{r}$ are the order of moments from first to fourth [57]. As well as k,l represents the lag-parameters. Hence, using the non-linear combinations of the moments, cumulant can be computed.

(13) $$C_{1}^{r}=m_{1}^{r}$$

(14) $$C_{2}^{r}=m_{2}^{r}\left(k\right)$$

(15) $$C_{3}^{r}=m_{3}^{r}\left(k,l\right)$$

(16) $$C_{4}^{r}=m_{4}^{r}\left(k,l,q\right)-m_{2}^{r}\left(k\right)-m_{2}^{r}\left(l-q\right)-m_{2}^{r}\left(q-k\right)-m_{2}^{r}\left(q\right)m_{2}^{r}\left(k-l\right).$$

Here $C_{1}^{r}$, $C_{2}^{r}$, $C_{3}^{r}$, and $C_{4}^{r}$ are the order of cumulants from first to fourth. In this work, we have computed second, third, and fourth order cumulants.

#### 7.1.11. Recurrence Plot (RP)

For the physiological signal in the time domain, RP can find hidden patterns which are not clearly identifiable [57]. Recurrence can be defined as the value of *k* and *l* dropped below the threshold value ϵ is known as recurrence. Assuming $r_{k}$ in an *L* dimensional space be the kth point. However, a dot is considered at (k,l) as the distance between the $r_{k}$ and $r_{l}$ is closer. When k=l, the recurrence plots are symmetric along the diagonal, as $r_{k}$ is close to $r_{l}$, then $r_{l}$ is close to $r_{k}$. Hence, a RP is R×R square of an array dots [57]. This may also be presented in time-related space as a R×R matrix. The yellow dot implies there has been a recurrence.

#### 7.1.12. Recurrence Quantification Analysis (RQA)

RQA is better choice for dynamical system used to measure the number and duration of recurrence. It helps to measure and analyze the recurrence plot of non-stationary physiological signal [57]. In the time domain, RQA evaluates the non-stationary and hidden periodicity of signals. The following RQA features are used in this work:

(a) Recurrence rate (RR), (b) Determinism (DET), (c) Entropy (ENT), (d) Laminarity (LMR) [57].

### 7.2. Results

The proposed work is performed on LRHT, HRHT and HC subjects. A total of 3694 ECG segments were obtained from SHAREE and PTB databases. The LRHT have 3172, HRHT have 442, and HC class have 80 ECG signal segments of 2 min duration.

An experiment is performed on MATLAB 2016b version 9.1.10 (licensed) and work station (personal computer) with Intel i7 processor, 16GB RAM, 1TB HD, and 4 GB graphics card. We have tried several non-linear features to obtain the optimum results. However, higher order spectral cumulant, bispectrum and recurrence quantitative analysis (RQA) yielded optimum results.

In addition to this, the optimum performance were obtained using the combination of HOS bispectrum, cumulant and RQA feature. A total of 9 bispectrum-based features are extracted and shown in Table 5. The detailed of RQA and HOS cumulant features are presented in Table 6 and Table 7 respectively.

**Figure 5:**
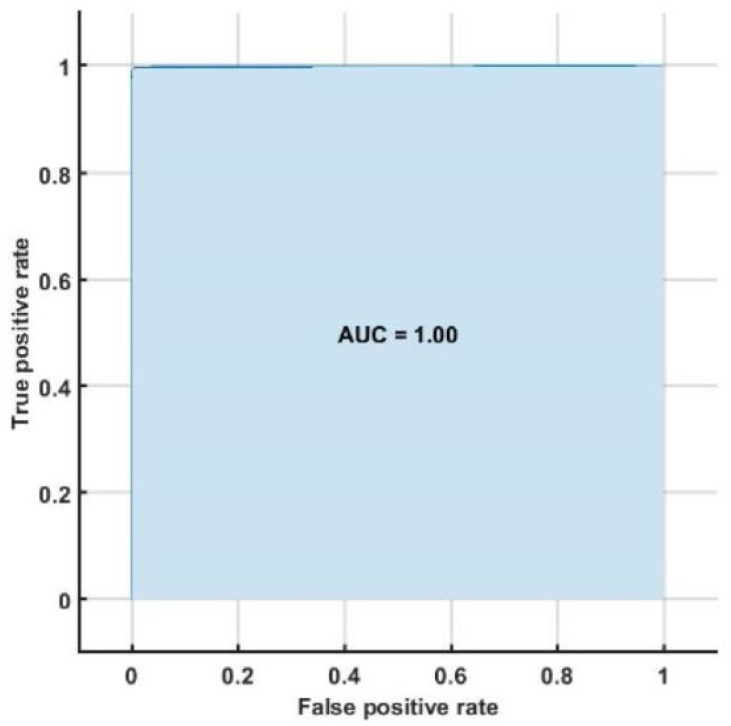
ROC plot obtained with SVM classifier.

The highest classification accuracy, sensitivity and specificity of 98.05%, 95.66%, and 96.58%, respectively are obtained using support vector machine classifier with ten-fold cross-validation strategy. Table 8 represents the confusion matrix obtained for SVM classifier using all bispectrum, cumulant and RQA features. Table 9 shows the performance measures obtained for each class using HOS bispectrum, cumulants and RQA features with SVM classifier. The highest AUC of 1.00 is obtained using SVM classifier (Figure 5).

**Figure 6:**
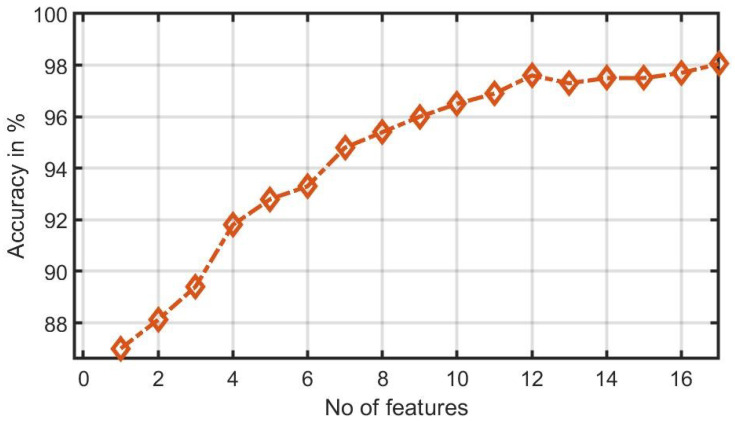
Graph of accuracy (%) versus combined features (bispectrum, cumulants and RQA).

**Figure 7:**
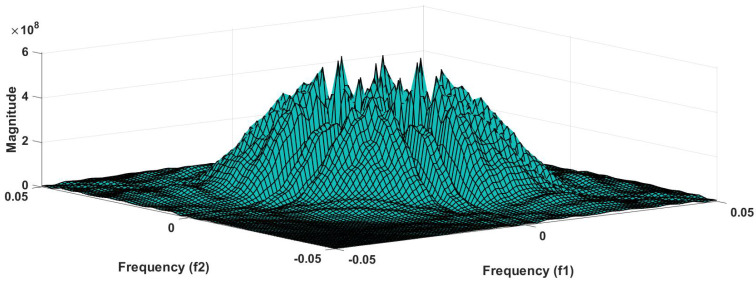
HC-bispectrum plot.

**Figure 8:**
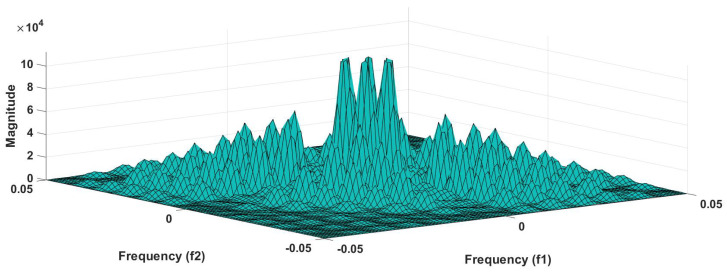
HRHT-bispectrum plot.

**Figure 9:**
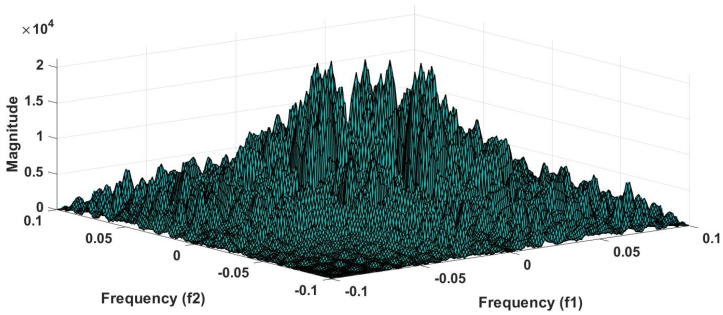
LRHT-bispectrum plot.

**Figure 10:**
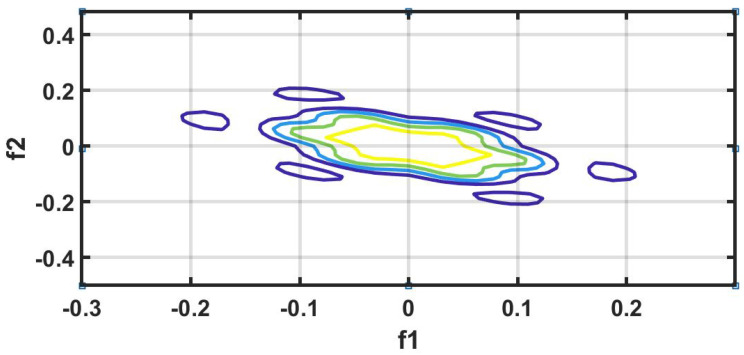
HC-contour plot.

**Figure 11:**
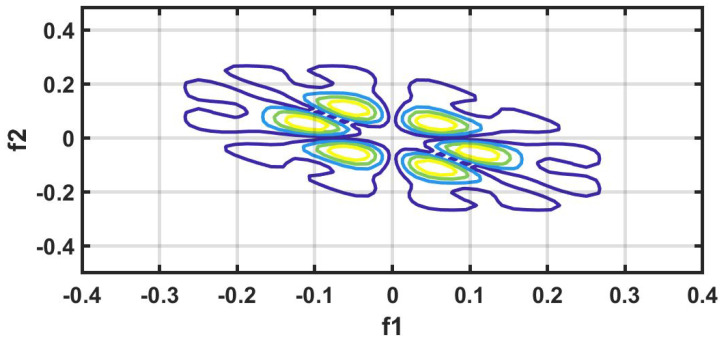
HRHT-contour plot.

**Figure 12:**
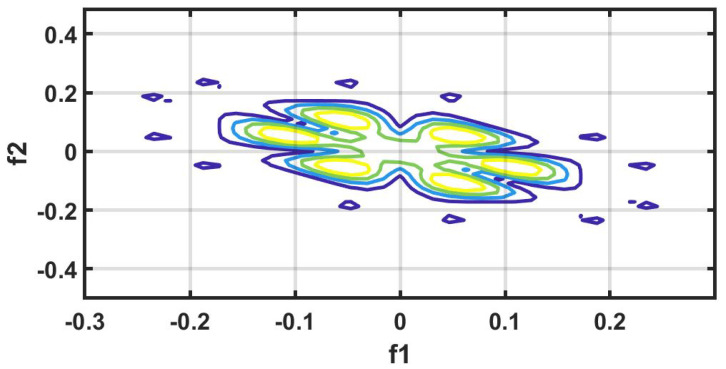
LRHT-contour plot.

**Figure 13:**
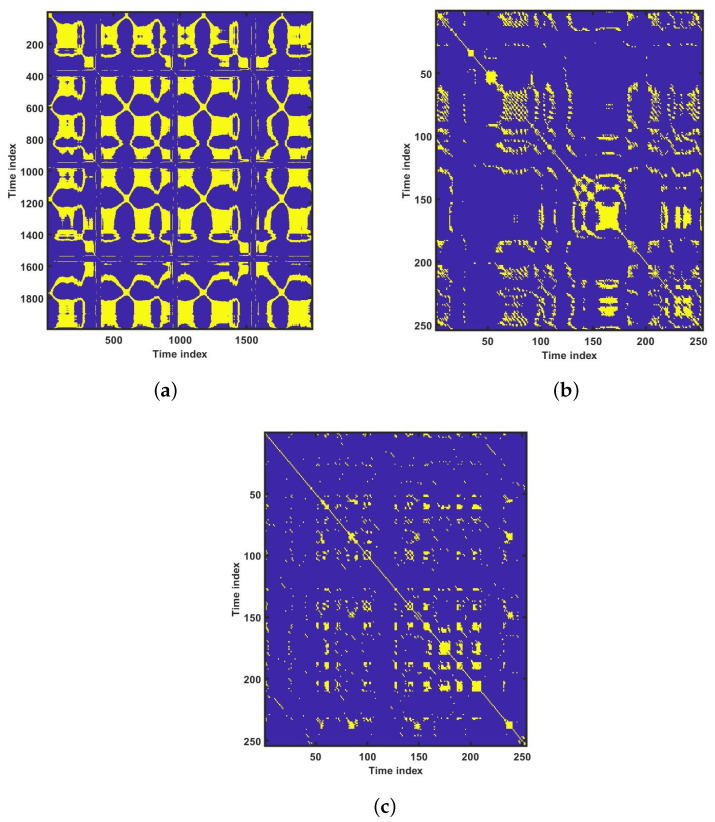
Recurrence plots for various groups: (**a**) healthy controls, (**b**) HRHT, and (**c**) LRHT.

Figure 6 shows the plot of accuracy % versus the number of features. HOS bispectrum magnitude and contour plots for HC, LRHT and HRHT class are shown in the Figure 7, Figure 8, Figure 9, Figure 10, Figure 11 and Figure 12. Figure 13 shows the recurrence plots for HC, LRHT, and HRHT classes. Summary of classification performance obtained using various combination of features is shown in Table 10.

## 8. Discussion

Table 2, Table 3 and Table 4 summarizes all studies using ECG, BCG, PPG, and HRV signals. It is also evident from Table 2 that the ECG-based computed aided diagnosis system obtained the highest area under receiver operating characteristics (AUC=1.00) performance compared to rest of methods. Moreover, Table 2 represents the highest classification accuracy of 99.99% using ECG signals.

Table 11 lists the summary of artificial intelligence (AI) techniques used to classify HT based on ECG and HRV signals. On the other hand, Table 2, Table 3 and Table 4 summarize the methods, features, subjects, results and type of databases that have been used to diagnose HT using HRV, ECG, BCG, and PPG signals. In both ECG and HRV signal-based studies, authors have used transformational approaches, converted time-domain signals into the frequency domain, extracted non-linear features and classified using SVM and KNN classifiers. The summary of automated systems developed for HT are as follows:

- Rajput et al. [8] developed an HDI accurately using ECG signals to stratify low-risk versus high-risk HT with a single numeric value.
- Poddar et al. [28] used HRV signals to classify HT and normal subjects using SVM classifier with 100% accuracy using 20 features. They have used a balanced data set of 56 normal and 57 HT subjects in their study.
- Rajput et al. [23] classified ECG signals into three classes (LRHT, HRHT, and HC) using features extracted from the five-level wavelet decomposition of ECG signals. They have obtained 99.95% classification accuracy using SeEn and WeEn features with unbalanced data set. Testing error is found to be only 3.26% with hold-out validation method.
- Soh et al. [24] developed a CNN architecture for the classification of normal and HT ECG classes and achieved an accuracy of 99.99%, sensitivity of 100% and specificity of 99.97%.

In this work, bispectrum based features obtained the highest classification accuracy of 96.5% among the nonlinear features. The summary of classification performance obtained using various combination of features is shown in Table 10.

It can be noted from Table 5 that bispectrum features are clinically significant and show clear difference between three classes. On the other hand, Hos cumulant order-three mean and standard values yielded the large difference among three class as mentioned in Table 7, hence it is useful for 1-D signal features extraction. However, RQA RR feature also comprising distinct difference in all three class.

It is well recognized that the bispectrum conserves the phase information [57]. Because of this property, it is used to examine quadratic nonlinear differences between various frequency components of ECG signals. Such interactions have been observed between different frequencies of three classes of ECG signals. This analysis may be useful for detecting changes in ECG signals. For a normal, LRHT, and HRHT ECG signal, the magnitude and its contour representation are shown in the Figure 7, Figure 8, Figure 9, Figure 10, Figure 11 and Figure 12. It can be noted from Figure 7, Figure 8, Figure 9, Figure 10, Figure 11 and Figure 12 that, these plots are unique and can be used to discriminate the class of the ECG signal (HC, LRHT, or HRHT). Similarly, Figure 13 shows the recurrence plots for HC, LRHT and HRHT ECG signals. These plots are unique and can also be used to differentiate the three classes. We have obtained the highest classification performance only using HOS bispectrum features without transforming the ECG signals (Table 10). The advantages of proposed study are: (i) many nonlinear features are employed which can be used for the classification. (ii) Proposed unique HOS bispectrum and recurrence plots for three classes. (iii) HOS-based features are more robust to noise.

In general, works conducted using ML and DL coupled with ECG signals have yielded the highest and optimum performance. The works done in Table 2, Table 3 and Table 4,^2^,^8^,^13^,^16^,^21^,^23^,^24^,^25^,^26^,^34^,^35^,^26^] have used public (open source) databases, while the rest of the studies have used private databases. This underscores the importance of public databases for computer aided diagnosis systems development.

Liang et al. [20,34,35] detected HT using PPG signal in three separate studies using public and private databases. They achieved a best classification F-score of 94.84%.

Liu et al. [21] diagnosed HT from BCG-derived HRV signal, and achieved highest classification accuracy of 84.4% using ML.

Such results demonstrate the effectiveness of transformation methods that combine nonlinear and entropy-based features. ML methods work well with balanced and smaller databases. The performance of ML models also depends on the features extracted and classifiers used.

In the future, we intend to use deep learning architectures to detect the HT ECG signals using large database [59]. The biggest challenge for this study is the availability of the large public database.

**Figure 14:**
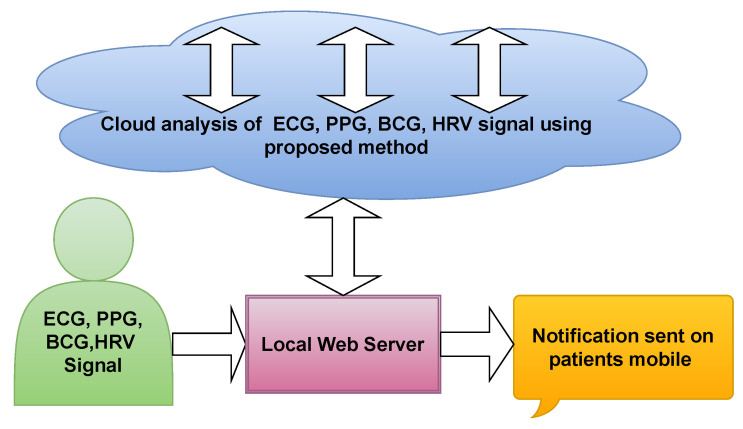
Proposed cloud based model.

Figure 14 illustrate the cloud based proposed model. Initially, the ECG, PPG, BCG, and HRV signal recorded from patients and stored in hospital database. The stored signals were sent to the cloud based model, where it is installed. The cloud based model analyze the provided data and detect the hypertension accurately. To the same, the results were revert from cloud to hospital. Hence, the Doctors can compare the results obtained by cloud based model as well as manually finding. Table 12 have all the list of abbreviation used in the paper.

## 9. Conclusions

We have reviewed many automated HT diagnosis methods using ECG and other physiological signals. Many ML models have been developed using nonlinear features and various classifiers. Few DL architectures have been proposed to detect HT ECG signals. Combined with low-cost wearable devices, such methods have the potential to monitor for continuous, non-intrusive cuffless and wireless remote BP. Such automated systems are reliable, accurate and can also be used to detect other cardiac ailments. It can be used in hospital intensive care units (ICUs) to aid the staff to alert the sudden rise in the BP of patients immediately and provide accurate treatment.

## Figures and Tables

**Table 1:** Classification of HT based on office blood pressure measurement [1].

Category	Systolic (mm Hg)		Diastolic (mm Hg)
Normal BP	<130	and	<85
High-normal BP	130–139	and/or	85–89
Grade 1 hypertension	140–149	and/or	90–99
Grade 2 hypertension	≥160	and/or	≥100

**Table 2:** All details used in HT diagnosis from ECG signal.

S No.	Author/Year	Signal	Feature	Method	Subject	Database	Results
1	Rajput et al. [2019] [8]	ECG	Signal fractal dimension and Log energy	Wavelet decomposition using FB, feature extraction, student-t test, developed index	139	SHAREE	100 % discrimination of LRHT, HRHT
2	Soh et al. [2020] [2]	ECG	18, non-linear	EMD is used to decomposed ECG signal up-to 5 level using IMF, feature extraction, student t-test and then used supervised KNN classifier	157	SHAREE, MIT-BIH	ACC = 97.70% , SEN = 98.90%, SPE = 89.10%
3	Rajput et al. [2020] [23]	ECG	SeEn and WlEn	Wavelet decomposition using FB, feature extraction, used EBT classifier to classify severity of HT	191	SHAREE, PTB	ACC = 99.95%, SEN = 98.64%, SPE = 99.91%, F1 = 97.3% AUC = 1
4	Liang et al. [2018] [35]	ECG, PPG	Ratio, Slope, Power area, waveform area, VPG and APG, Time span, PPG amplitude, PAT Feature	Classification of HT	121	MIMIC	SEN = 94.26%, SPE = 96.17%, F1 = 94.84%
5	Soh et al. [2020] [24]	ECG	Total 1507	Classification using CNN, DL model	157	SHAREE, MIT-BIH	ACC = 99.99%, SEN = 100%, SPE = 99.97%
6	Jain et al. [2020] [25]	ECG	11 layer CNN	Classification using CNN, DL model	191	SHAREE	ACC = 99.68%
7	Present study	ECG	HOS, bispectrum, Cumulant, RQA	Direct feature extraction and classification	191	SHAREE, PTB	ACC = 98.05%, SEN = 95.66%, SPE = 96.58%

**Table 3:** All details used in HT diagnosis from HRV and BCG signals.

S No.	Author/Year	Signal	Feature	Method	Subject	Database	Results
1	Melillo et al. [2015] [16]	HRV	PP ( SD1 and SD2), CD, DFA (features: Alpha1,Alpha2), and RP and HRV	Statistical analysis	139	SHAREE	ACC = 85.7%, SEN = 71.4%, SPE = 87.8%
2	Ni et al. [2019] [13]	HRV	18 HRV multidimensional features	Wavelet transform,	139	SHAREE	AUC = 0.95
3	Y.song et al. [2015] [33]	HRV, BCG	HRV time and frequency domain feature and DFA	EEMD, data-mining, DFA	18	Private	ACC = 92.3%
4	Poddar et al. [2014] [37]	HRV	Nonlinear parameters of PP, ApEn and SeEn and HRV time and frequency domain feature	Classification of HRV	113	Private	ACC = 100%, SEN = 100%, SPE = 100%
5	Natrajan et al.[2014] [18]	HRV	HRV feature	Statistical analysis using SPSS	60	Private	HRV reduce in HT subjects
6	Ni et al. [2017] [30]	HRV	ApEn and SeEn and HRV time and frequency domain feature	Classification of HRV signal	24	Private	ACC = 93.3%
7	Poddar et al. [2019] [29]	HRV	HRV time and frequency domain feature	Classification of HRV	185	Private	ACC = 96.7%
8	Koichub et al. [2018] [38]	HRV	HRV time and frequency domain feature, CD	Statistical analysis	56	Private	HRV decreased in HT group
9	Tejera et al. [2011] [27]	HRV	LZ, and SeEn, HRV time and frequency domain feature	ANN	568	Private	SPE = 90% , AUC = 0.98
10	Mussalo et al. [2008] [32]	HRV	HRV time and frequency domain feature	Statistical analysis using SPSS	97	Private	LF, HF power decrease in SEHT group
11	Liu et al. [2019] [21]	HRV, BCG	HRV time and frequency domain feature, SeEn, DFA, BCG fluctuation features	Classification, feature extraction, selection, identification of HT	128	Open source	ACC = 84.4%, PRE = 82.5%, REC = 85.3%
12	Kublanov et al. [2017] [31]	HRV, ECG	CWT, HRV feature	Classification of HT	71	Private	Score = 91.33% ± 1.73
13	Alkhodari et al. [2020] [26]	HRV	HRV feature	Low and high-risk HT	139	SHAREE	ACC = 97.08%

**Table 4:** All details used in HT diagnosis from PPG signal.

S No.	Author/Year	Signal	Feature	Method	Subject	Database	Results
1	Liang et al. [2018] [34]	PPG	CWT	Classification using Pre-trained CNN (GoogLeNet, 144 layer)	121	MIMIC	F1-score = 92.55%
2	Liang et al. [2018] [20]	PPG	Ratio, Slope, Power area, waveform area, VPG and APG, Time span, PPG amplitude	Classification of HT	124	Private	PP = 100%, SE = 85.71%, F1-score = 92.31%
3	Lan et al. [2018] [15]	PPG, HRV	HRV time and frequency domain feature	Data mining	43	Private	ACC = 85.47%, SPE = 83.33%, PRE = 92.11%
4	Ghose et al. [36]	PPG , HRV	Mean, SD, min and max, HRV time and frequency domain feature	Classification of HT	20	Private	F1-score = 83%

**Table 5:** Summary of bispectrum features (mean ± standard deviation) values obtained for three classes.

Bispectrum	LRHT	HRHT	HC
$NBE_{1}$	0.927 ± 0.044	0.866 ± 0.076	0.582 ± 0.237
NBSE	0.707 ± 0.161	0.538 ± 0.181	0.097 ± 0.11
$WCOB_{1m}$	3324 ± 1369	2297 ± 1170	714 ± 820
$WCOB_{2m}$	1524 ± 733	846 ± 620	333 ± 387
$M_{1}$	1.9 × $10^{8}$ ± 1.4×$10^{7}$	1.8 ×$10^{8}$ ± 1.3 × $10^{7}$	3×$10^{8}$ ± 1.4×$10^{7}$
$M_{2}$	40,873 ± 2946	38,718 ± 2743	63,642 ± 2803
$M_{3}$	9.7×$10^{7}$ ± 7×$10^{6}$	8.9×$10^{7}$ ± 6×$10^{6}$	1.5×$10^{8}$ ± 7×$10^{6}$
mAmp	4.3×$10^{9}$ ± 4× $10^{10}$	9.6×$10^{9}$ ± 3.5× $10^{9}$	7.7×$10^{14}$ ± 1.8× $10^{15}$
$Ph_{e}$	3.58 ± 0.00028	3.58 ± 0.00048	3.56 ± 0.063

**Table 6:** Summary of RQA features (mean ± standard deviation) values obtained for three classes.

RQA	LRHT	HRHT	HC
RR	8×$10^{-4}$ ± 7×$10^{-5}$	9× $10^{-4}$ ± 8 ×$10^{-5}$	5× $10^{-4}$ ± 1× $10^{-5}$
DET	0.375 ± 0.0928	0.483 ± 0.138	0.508 ± 0.0972
ENT	0.486 ± 0.112	0.628 ± 0.190	0.662 ± 0.143
LMR	2.448 ± 0.407	2.685 ± 0.868	2.748 ± 0.289

**Table 7:** HOS cumulant second, third, and fourth order features computed (mean ± standard deviation) values obtained for three classes.

HOS Feature	LRHT	HRHT	HC
Cumulant${}_{2}$	125.23 ± 432.91	93.42 ± 176.32	8×$10^{5}$ ± 2× $10^{6}$
Cumulant${}_{3}$	17.232 ± 4269.5	−1111.3 ± 4534	−8 ×$10^{8}$ ± 7×$10^{9}$
Cumulant${}_{4}$	92,476 ± 1 ×$10^{6}$	1× $10^{5}$ ± 5 ×$10^{5}$	3× $10^{12}$ ± 6 ×$10^{13}$

**Table 8:** Confusion matrix with SVM classifier using HOS bispectrum, cumulant and RQA features.

	HC	HRHT	LRHT
HC	79	0	1
HRHT	0	393	49
LRHT	0	22	3150

**Table 9:** Performance parameters obtained using HOS bispectrum, cumulants and RQA features with SVM classifier.

Class	Accuracy%	Sensitivity%	Specificity%	F1-Score%
HC	99.97	98.75	100	99.37
HRHT	98.07	88.87	99.32	91.71
LRHT	98.05	99.30	90.42	98.87

**Table 10:** Summary of classification performance obtained using various combination of features.

S.No	Feature	Accuracy %	AUC	Classifier
1	HOS cumulant order2,3,4	90.2	0.99	EBT
2	HOS bispectrum	96.3	0.99	KNN
3	RQA	91.0	1.00	EBT
**4**	**bispectrum, Cumulant, RQA**	**98.05**	**1.00**	**SVM**
4	SeEn	84.3	0.74	TREE
5	WeEn	88.0	0.96	TREE
6	ApEn	81.8	0.94	EBT
7	ReEn	78.9	0.88	EBT
8	SeEn, WeEn, ApEN, ReEn	89.1	0.97	EBT
9	SLFD	87.1	0.96	SVM
10	HE	87.8	0.92	SVM
11	LLE	82.4	0.86	NB
13	SLFD, HE, LLE,	88.1	0.97	EBT
15	LOGE	86.4	0.94	TREE
16	SeEn, WeEn, ApEN, ReEn, SLFD, HE, LLE, LOGE	95.5	0.99	EBT

**Table 11:** Summary of works carried out on automated detection of HT diagnosis.

S No.	Author/Year	Type of ML	Classifier
1	Soh et al. [2020] [2]	Supervised ML	KNN
2	Melillo et al. [2015] [16]	Supervised ML	AB, NB, RF, SVM
3	Ni et al. [2019] [13]	Supervised ML	SVM,RF,NB
4	Song et al. [2015] [33]	Supervised ML	SVM, RF, KNN
5	Poddar et al. [2014] [37]	Supervised ML	SVM
6	Ni et al. [2017] [30]	Supervised ML	Linear SVM
7	Poddar et al. [2019] [29]	Supervised ML	SVM, KNN
8	Tejera et al. [2011] [27]	ANN	ANN
9	Rajput et al. [2020] [23]	Supervised ML	KNN, SVM, TREE, and EBT
10	Liu et al. [2019] [21]	Supervised ML	SVM, DT, NB
11	Liang et al. [2018] [34]	DL	CNN, GoogLeNet
12	Liang et al. [2018] [20]	Supervised ML	LDA, SVM, KNN, LR
13	Liang et al. [2018] [35]	Supervised ML	AB, KNN, EBT, LR
14	Lan et al. [2018] [15]	Semi-supervised learning	-
15	Ghose et al. [36]	Supervised ML	AB, KNN, EBT, DT, RF, NB, SVM
16	Kublanov et al. [31]	Supervised ML	LDA, SVM, KNN, NB, DT
17	Soh et al. [2020] [24]	DL model	CNN
18	Jain et al. [2020] [25]	DL model	
19	Alkhodari et al. [2020] [26]	ML	RUSBOOST, TREE, SVM
20	Present study	Supervised ML	KNN, EBT, SVM

**Table 12:** Abbreviation used in the review study.

Abbreviation	Full Form	Abbreviation	Full Form
SLFD	Signal fractal dimensions	LOGE	Log energy
LLE	Largest Lyapunov Exponent	HOS	Higher order spectral
OGWB	Orthogonal wavelet filter bank	
HT	Hypertension	SBP	Systolic blood pressure
HRV	Heart rate variability	DBP	Diastolic blood pressure
ECG	Electrocardiography	DWT	Discrete Wavelet Transform
PPG	Photoplethysmography	BCG	Ballistocardiogram
LVH	left ventricular hypertrophy	VG	ventricular gradient
PPG	Photoplethysmography	HDI	Hypertension diagnosis index
ML	Machine learning
DL	Deep Learning	ANN	Artificial Neural Network
CNN	Convolution neural network	RNN	Recurrent Nural Network
SVM	Support vector machine	KNN	K-nearest neighbour
CWT	Continuous Wavelet Transform	FFT	Fast Fourier transform
ANOVA	Analysis of variance	ROC	Receiver operating characteristics
EBT	Ensemble Bagged Tree	AB	Ada boost
LR	Logistic Regression	NB	Navy Bayes
RF	Random Forrest	LRA	Linear Regression Analysis
SeEn	Sample entropy	ApEn	Approximate entropy
ReEn	Reny entropy	WlEn	Wavelet entropy
DFA	Detrended fluctuation analysis	CD	Correlation Dimension
LZ	Lempel-Ziv complexity	RC	Recurrence Plot
PP	Poincare plot	EMD	Empirical Mode Decomposition
VPG	Velocity plethysmogram	APG	Acceleration plethysmogram
PAT	Pulse arrival time	INVD	Inverse dower
ACC	Accuracy	SPE	Specificity
SEN	Sensitivity	PRE	Precision
REC	Recall	AUC	Area under the curve
PPV	Positive predictive value	NPV	Negative Predictive Value
SPSS	Statistical Package for the Social Sciences	MANOVA	Multivariate analysis of variance MANOVA
PRISMA	Preferred reporting items for systematic reviews and meta-analyses	HRHT	High-risk hypertension
RUSBOOST	random under-sampling boosting	KNN	K-nearest neighbour
HC	Healthy control	LRHT	Low-risk hypertension
DT	Decision tree	LDA	Linear Discriminate analysis

## Data Availability

Data sharing not applicable.
